# Time Above All Else: Pharmacodynamic Analysis of Β-Lactams in Critically Ill Patients

**DOI:** 10.1017/ash.2021.135

**Published:** 2021-07-29

**Authors:** Katherine Landmesser, David Burgess, Justin Clark

## Abstract

**Background:** Despite the development of new β-lactam agents, gram-negative resistance continues to be an increasing concern in the healthcare setting. The understanding and optimizing antimicrobial pharmacokinetics and pharmacodynamics are essential to enhance activity of appropriate therapy, improve clinical outcomes, and reduce the development of resistance. **Methods:** A pharmacodynamic analysis was performed for 4 β-lactams (aztreonam, cefepime, piperacillin/tazobactam, and meropenem) and 14 dosage regimens as either intermittent bolus (IB) or prolonged infusion (PI) against 7 gram-negative pathogens: *Klebsiella pneumoniae*, *K. oxytoca*, *Escherichia coli*, *Enterobacter cloacae*, *E. aerogenes*, *Acinetobacter baumannii*, and *Pseudomonas aeruginosa*. Unit-specific minimum inhibitory concentration (MIC) distribution data were generated using antibiogram data over a decade for 4 intensive care units within our institution: medical ICU, cardiovascular ICU, surgical ICU, and neurosurgical ICU. Published pharmacokinetic parameter estimates in critically ill patients, combined with this MIC distribution data, were utilized to perform Monte Carlo simulations for each antimicrobial regimen. The percentage of time for which the unbound concentration of antibiotic remained above the MIC (ƒT>MIC) was utilized as the pharmacodynamic target for each agent: 40% ƒT>MIC for meropenem, 50% ƒT>MIC for piperacillin/tazobactam, 60% ƒT>MIC for aztreonam, and 70% ƒT>MIC for cefepime. Regimens were modeled using Oracle Crystal Ball software to determine the likelihood of achieving >90% probability of target attainment (PTA). Because resistance rates were significantly higher for *P. aeruginosa* and *A. baumannii*, cumulative PTAs for *K. pneumoniae*, *K. oxytoca*, *E. coli*, *E. cloac*ae, and *E. aerogenes* were analyzed separately to determine the relative PTA for Enterobacterales in each ICU. **Results:** No intermittent infusion regimens of piperacillin/tazobactam, aztreonam, or cefepime achieved >90% PTA for any organism. Piperacillin/tazobactam 4.5 g infused over 4 hours (PI q6h) and aztreonam 2 g PI q6h failed to achieve adequate PTA for Enterobacterales with only 84% and 85% PTA, respectively. For Enterobacterales, the only regimens to achieve >90% PTA included cefepime 2 g infused over 3 hours (PI q8h) and meropenem 1g IB q8h with 95% and 99% PTA, respectively. Meropenem 2 g PI q8h was the only regimen capable of achieving >90% PTA for both *A. baumannii* and *P. aeruginosa* with 97% and 92% PTA, respectively. **Conclusions:** Although utilization of high doses and prolonged infusions dramatically improve the pharmacodynamics of β-lactam therapy, the only regimen capable of achieving adequate PTA for all organisms analyzed was meropenem 2g PI q8h. To reduce carbapenem use, combination therapy may be considered for critically ill patients receiving aztreonam, cefepime, or piperacillin/tazobactam for empiric treatment of gram-negative infections.

**Funding:** No

**Disclosures:** None

